# Bioinformed idiographic symptom networks to identify neurobiological predictors of affective-state transitions in bipolar disorder

**DOI:** 10.1162/IMAG.a.1327

**Published:** 2026-08-03

**Authors:** Siraj J. Lyons, John Foley, Olivia Cook, Brendan E. Depue

**Affiliations:** Department of Psychological & Brain Sciences, University of Louisville, Louisville, KY, United States; Surgical Theater, Beachwood, OH, United States; Department of Anatomical Sciences & Neurobiology, University of Louisville, Louisville, KY, United States

**Keywords:** bipolar disorder, biological mechanisms, idiographic network analysis, neuroscience, mood disorders

## Abstract

Bipolar disorder remains an understudied psychiatric condition. Research has been focused on neurobiological mechanisms with the hope of identifying unique markers distinguishing bipolar disorder from similar conditions (i.e., major depressive disorder and schizophrenia), in addition to neurocognitive mechanisms driving affective state transitions. While these investigations continue to gain popularity, the current literature does not present a strong account for a neurobiological cause of affective state transitions and document significant heterogeneity between individuals. We argue that current difficulties with identifying neurobiological associations of affective state transitions can be targeted by incorporating idiographic symptom network analyses, a statistical and methodological tool more commonly used within the behavioral psychopathology literature, into the neurobiological study of bipolar disorder. Idiographic symptom networks allow the modeling of temporal relationships between symptoms and behavior at a finer temporal resolution compared to standard longitudinal analyses. As such, collecting many within-subject neurological measures samples alongside ecological momentary assessments indexing transient mood and cognitive functioning can provide an opportunity to identify potential neurobiological drivers of bipolar disorder symptoms and affective state transitions. The perspective explores current methodological designs, their associated strengths and limitations, in addition to the clinical utility with adopting idiographic network analyses within clinical neuroscience research.

## 1 Introduction

Bipolar disorder (BD) is a major psychiatric condition impacting 2.4% of adults worldwide (Merikangas et al., 2011), subsequently increasing risks of physiological disease (Forty et al., 2014; Warner et al., 2023), substance use disorders (Cerullo & Strakowski, 2007), and suicide (Dong et al., 2020) for millions of people. Individuals living with bipolar disorder cycle between depression and (hypo)mania, interspersed by periods of symptom remission (American Psychiatric Association, 2013; World Health Organization, 2019). While these affective states are defined to be categorically distinct from each other, individuals often experience states with co-existing (hypo)manic and depressive symptoms (Cassano et al., 2004; Zahn, 2025). In addition, individuals diagnosed with BD experience a greater risk of suicide during depressive affective states, particular those that present with (hypo)mania symptoms (Arnone et al., 2024). The neurobiological underpinnings have been sought since the condition was empirically delineated in the early 1900s (Kraepelin, 1921). At present, significant progress has been achieved, particularly with the advent of multi-site consortia constructing large-scale datasets. Yet, a neurobiological explanation of *affective state transitions* between depression, (hypo)mania, and euthymia remains elusive. At present, it is established that BD is associated with significant inter-individual heterogeneity that creates difficulty in identifying generalizable neurobiological features (Craddock & Sklar, 2013). The current perspective article presents the utility of integrating research methodology inspired by the behavioral psychopathology literature and the use of idiographic symptom network modeling. Typical research in human neuroscience investigates group-wise neurobiological characteristics within a single timeframe (i.e., cross-sectional) or across a few sessions in the timeframe of months or years (i.e., longitudinal). Idiographic symptom network modeling uses ecological momentary assessment to acquire multiple, daily samples to construct individual-specific temporal relationships between distinct symptoms. In short, we argue that increasing within subject neuroimaging data, akin to idiographic symptom network modeling, may better account for heterogeneity and subsequently provide more precise neurobiological linking to affective and behavioral symptomatology via idiographic network analysis, therefore establishing potential neurobiological causes of affective trait transitions in BD.

## 2 Current Neurobiological Literature of Bipolar Disorder

Within the past decade, focus on large-scale datasets through meta- and mega-analyses has increased to bolster attempts at identifying neurobiological phenotypes of BD through structural and functional MRI. In brief, measures from structural MRI reveal nonspecific reductions in cortical thickness across both cerebral hemispheres (Hibar et al., 2018). Moreover, BD is significantly associated with reduced indices of white-matter integrity, particularly the corpus callosum and cingulum (Favre et al., 2019). Furthermore, a multi-site investigation including several thousand participants found that sensorimotor and basal ganglia morphometry were the most important features distinguishing BD from major depression (Nunes et al., 2020). Functional neuroimaging investigations have aimed to identify trait- and affective state-specific neurocognitive mechanisms of BD. In particular, activity within the amygdalo-hippocampus complex is enhanced across affective states, including euthymia (Mesbah et al., 2023). In contrast, frontal and parietal activity elicited by working memory or reward processing tasks appeared specific to affective state (Mesbah et al., 2023). Frontoparietal dynamics are particularly interesting to researchers as they contribute to executive functioning (Marek & Dosenbach, 2018; Miller & Cohen, 2001), one core executive function thought to underpin cognitive and affective symptomatology in BD (Bora, 2018). In a recent meta-analysis, motoric and motivational inhibition were associated with distinct activation patterns (Chan et al., 2023). Motoric inhibition underpinned by frontal, insular, and thalamic regions is more sensitive to the emotional content of environmental cues among individuals with BD in contrast to healthy individuals without a psychiatric condition. In addition, Chan et al. (2023) report motivational inhibition indexed by delay discounting revealed enhanced activation across the orbitofrontal cortex and striatum when selecting immediate rewards compared to delayed, yet greater rewards. Furthermore, a considerable number of individuals diagnosed with BD have taken at least one psychotropic medication (Weinstock et al., 2014), which promotes neuroanatomical and neurofunctional changes overlapping with probable neurobiological signatures of BD pathophysiology (Abé et al., 2023; Hibar et al., 2018), thus presenting as a significant confounding variable and many neuroimaging studies are confounded with such effects (Hafeman et al., 2012; Philip et al., 2008).

While cross-sectional studies account for the majority of research, longitudinal studies following individuals across affective states exist within the literature, albeit sparsely. One study investigating morphometric changes over 6 years revealed cortical thinning within the prefrontal cortex that linearly scaled with the number of manic episodes (Abé et al., 2023). It has been reported that dynamic co-activation of the amygdala and sensorimotor network is more frequently associated with mania while amygdala co-activation with the default mode network is associated with depression (Rey et al., 2021). Critically, the degree of functional coupling scaled linearly with symptom severity in both affective states, presenting a potential brain-behavior relationship across affective states within each individual. While promising, more work is necessary to establish the robustness of these longitudinal findings.

Broadly, the synthesis of recent neuroimaging studies generally reveals the importance of salience (Hibar et al., 2016; Perry et al., 2019), sensorimotor representation (Abé et al., 2023; Hibar et al., 2018), and executive functioning (Hibar et al., 2018) when individuals with BD are actively involved with their environment. However, it remains uncertain if these neurobiological features are inducing affective state transitions or simply represent a current affective state. Importantly, general mood states are highly transitory and fluctuate throughout the day. For instance, negative events affecting mood within a particular day can predict suicidality (King et al., 2024). Indeed, recent meta-analyses suggest that new research paradigms are needed to better our chances at identifying the neurobiological underpinnings of BD and affective state transitions (Jones et al., 2025; Parker & Ching, 2025). Thus, we argue that the implementation of multiple within-subjects sampling using both neuroimaging-derived and behavioral affective/cognitive measures will improve the detection of unique neurobiological predictors of affective state transitions.

## 3 Idiographic Networks

### 3.1 What are idiographic networks?

**Fig. 1.**
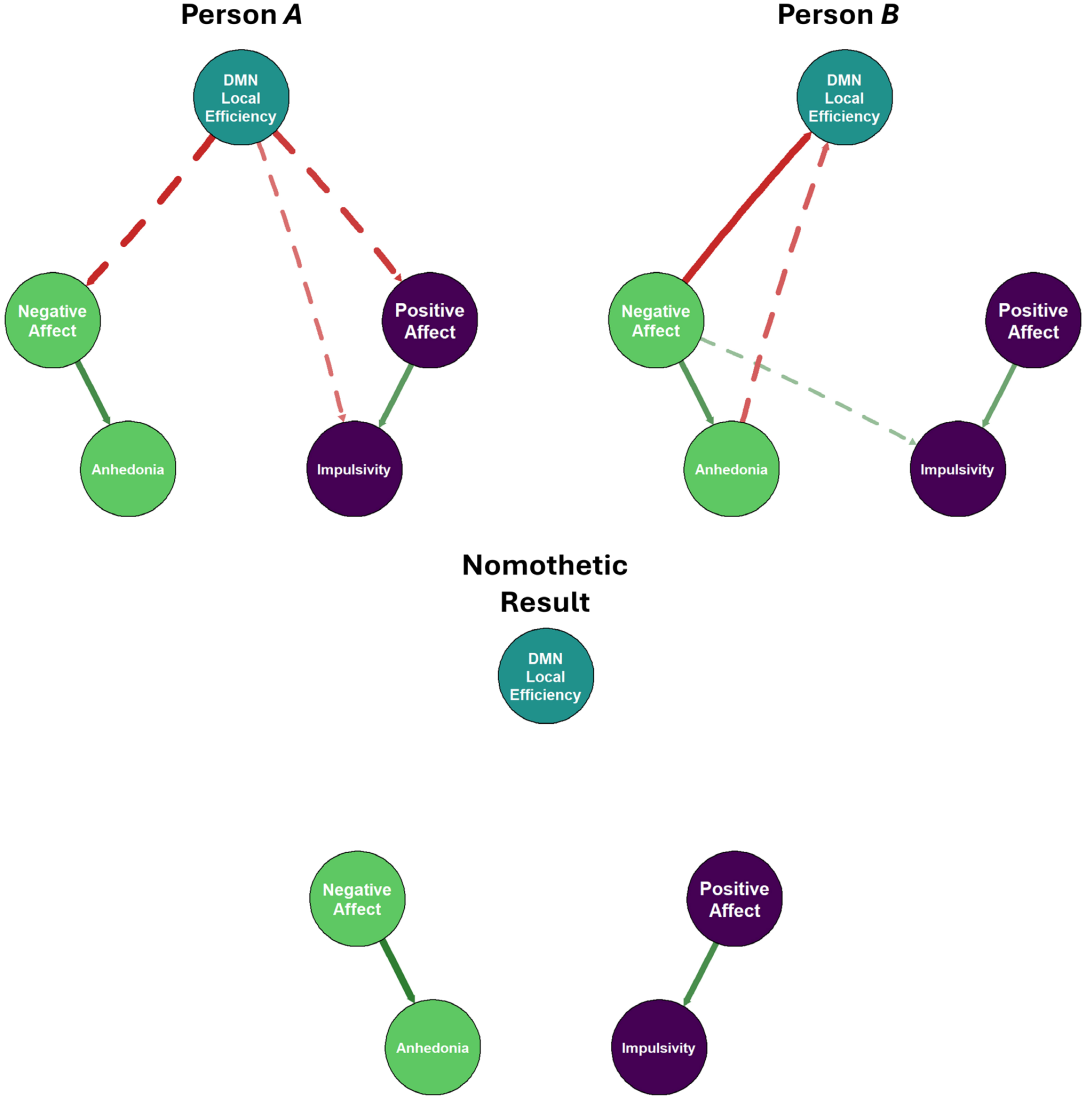
Idiographic networks identify unique associations between variables within individuals, allowing inter-individual variability (top panel). Such modeling is advantageous for investigating heterogeneous populations with complex symptomatology like bipolar disorder. A simple, fictitious model is depicted representing relationships between depressive (i.e., sadness and anhedonia in green) and manic (i.e., happiness and impulsivity in purple) symptoms with a neurobiological measure such as the local efficiency of the default mode network. Positive and negative relationships between variables, depicted by green and red arrows respectively, can be modeled within contemporaneous (solid lines) or temporally delayed (dashed arrows) contexts. Within this example, idiographic models can be used to explore mixed features, as evident within *Person A* who exhibits a lagged relationship between sadness and impulsivity. However, within this example, the shared features identified through traditional nomothetic analyses would only indicate the positive relationships between affective-state congruent symptoms (bottom panel).

A considerable proportion of behavioral and neuroimaging research fall underneath the category of *nomothetic* investigations, which seeks to observe general laws within the psychological sciences. In other words, nomothetic research constructs models generated from group-summarized datasets, and then is applied downward onto the individual. In recent years, viewing psychopathology as systems of dynamically interacting symptom clusters that carry significant inter-person variability has become increasingly popular (Fried et al., 2017; Öngür & Paulus, 2025) (see Fig 1 for a visual representation). Calls for integrating more person-centric data into psychological sciences have persisted since the early 2000s (Barlow & Nock, 2009; Molenaar, 2004), and continue to grow as the role of biological rhythms and their influence on brain structure and function become better understood (Mattoni et al., 2026; Vinci-Booher et al., 2025). Symptom-specific analysis, initially pioneered by Jaspers (1913), is well suited to be investigated within an individual-specific, or *idiographic*, framework to uncover unique trajectories and their temporal relationships are well suited to address research questions that emerge from this perspective. By modeling individual-specific symptom interactions, it is possible to identify particular symptoms that act as the “core” of one’s affective state (i.e., high centrality), or symptoms that influence other symptoms (i.e., bridging). Thus, we argue that future investigations targeting neurobiological predictors of BD and affective transitions ought to include idiographic methodologies.

### 3.2 Psychopathology under the idiographic perspective

A wide-range of psychopathologies have been investigated using idiographic modalities, including obsessive-compulsive disorder (Ong et al., 2024), eating disorders (Harris et al., 2025; Ralph-Nearman et al., 2024), major depressive disorder (Kullar et al., 2024), and BD (Bos et al., 2022; Curtiss et al., 2019; Kullar et al., 2024; Mesbah et al., 2024; Zavlis et al., 2023). For the purpose of the current commentary, we use bipolar disorder as a specific example. Mesbah et al. (2024) used dynamic time warping, which nonlinearly aligns two (or more) timeseries, of momentary assessments collected over a time period of 3–6 months, revealing that (hypo)manic symptoms evolve into dysphoric (hypo)mania, similar to the presentation of both positive and negative symptomatology, and increases in lethargy subsequently increase suicidal ideation (Mesbah et al., 2024). Idiographic network modeling has also been used to reveal interactions between positive and negative symptomatology. Among healthy people without psychopathology, positive and negative emotions possess an inverse relationship. Yet, in bipolar disorder, it appears that positive and negative emotions may upregulate each other (Curtiss et al., 2019). As such, identifying temporally connected positive and negative emotions may present a foundation for affective state transitions to occur. In another investigation, sleep, anhedonia, impulsivity, and suicidal ideation appear to act as”bridge” symptoms between these two states (Zavlis et al., 2023). Symptoms bridging affective states could be monitored over time to investigate potential warning signs of impending affective transition (Bos et al., 2022). According to a recent review presenting the neurocognitive perspective of BD, it will be of interest to investigate whether these bridges are underpinned by dopaminergic activity that drives anger and irritability (Zahn, 2025). While idiographic investigations within the BD literature is emerging, it is clearly demonstrated that it is a useful methodological tool for understanding affective and cognitive trajectories associated with the condition.

### 3.3 Using biological measures within idiographic analyses

While the majority of idiographic research studying psychopathology report exclusively on self-report or clinician-evaluated measures, only a few investigations have incorporated physiological measures to predict clinical outcomes. For instance, Ralph-Nearman et al. (2024) observed that continuous measures of heart rate, electrodermal activity, and peripheral skin temperature predict eating disorder behaviors outside of a laboratory setting. Heart rate activity at the final minute of completing a mathematical assessment in a laboratory setting predicts increasingly negative post-task appraisals (Quigley et al., 2002). Work by Bylsma et al. (2024) used physiological activity (e.g., heart rate variability) to understand its relationship with both emotional experiences throughout the day and emotion regulation. The authors observed that utilizing avoidant regulatory strategies throughout the day predicted increased heart rate variability. However, we note that the authors collected cardiac data only once while assessing emotional variables multiple times per day. Additionally, these investigations did not construct networks to determine variables connecting symptom clusters with physiology. Yet, network research is expected to grow in the future. Currently, there is an increasing desire to incorporate biological measures into idiographic modeling in other fields such as affective sciences (Garzón-Partida et al., 2025; Hoemann et al., 2023) and personality disorders (Lans et al., 2025). Such efforts carries utility for both clinical applications to enhance the personalization of treatment interventions. Moreover, basic research could still benefit using idiographic network analyses by using temporal modeling to identify potential neurobiological features predicting future affect and affective state transitions to test using large-scale neuroimaging studies.

In recent years, it has become more popular to combine MRI investigations with ecological momentary assessment to track day-to-day changes in cognitive function and mood (Gadassi-Polack et al., 2024; Stange et al., 2019). For instance, functional connectivity between the subgenual anterior cingulate cortex and dorsal anterior cingulate cortex predicts inflexibility of sadness and situational avoidance in both major depressive disorder and healthy controls (Schwartz et al., 2019). While none of the identified investigations in Stange et al. (2019) collected multiple MRI samples to temporally track affective symptoms, these studies indicate that neurobiological measures, in addition to cardiophsyiology, can predict transitory aspects of mood in daily life. However, a dataset presented by Filevich et al. (2017) represents an emerging ability to associated time-variable MRI measures with experiential momentary analysis (EMA) measures of mood and cognition. Indeed, one such study reported an association between the temporal variability of amygdalo-hippocampal, insular functional connectivity and affect (Racicot et al., 2024). Of interest, Group Iterative Multiple Model Estimate (GIMME) and Hidden Markov Models are both techniques used within EMA investigations to identify temporal relationships in behavior and affect already present within the neuroimaging literature (Beltz & Gates, 2017; Eavani et al., 2018; Wright et al., 2019). These approaches are conceptually amenable to increasing personalization of modeling brain-behavior relationships through idiographic network analyses.

### 3.4 Limitations of idiographic research

No methodology is without limitations. While much of the human neuroscience research is limited by the number of participants enrolled in a study, idiographic methods are more concerned with the number of assessments that are present within the dataset (Kuper et al., 2025). At present, a total of 75–100 observations appear necessary to provide power to effectively analyze the interactions between 6 variables (Mansueto et al., 2023). Subsequently, idiographic research is inherently more resource demanding for both the participants and the research team conducting the investigation, creating important hurdles to overcome. Consequently, it can prove difficult to collect neurobiological data, such as structural and functional MRI, within a reasonable timeframe to accurately determine a temporal relationship between biology and symptomatology. Additionally, it is difficult to forecast which of a multitude of variables will be critical to assess, which increases the degrees-of-freedom inherent to any analyses. Biologically-informed idiographic research will also be susceptible to recruitment difficulties and attrition experienced by standard clinical investigations (Astill Wright et al., 2025). Yet, it remains feasible to complete such research recruiting individuals with BD (Zavlis et al., 2023), and researchers can adopt strategies that improve participant retention like financial compensation, flexibly moulding research designs to individual participants, and establishing authentic and rich interpersonal rapport with participants (Astill Wright et al., 2025; Poongothai et al., 2023).

## 4 Recommendations

First, future work can develop coarse neurophysiological models relying on established idiographic methodology. Here we discuss: 1) appropriate dynamic peripheral temporal physiological measures, 2) dynamic functional neuroimaging measures, 3) simultaneous peripheral and neural recording, and lastly 4) incorporation of both into idiographic symptom network modeling.

### 4.1 Peripheral physiology

Early development of idiographic biological predictors of affective state transitions could take advantage of the relative simplicity of these measures before increasing granularity by exploring neurobiological structure and function using MRI. Ambulatory measures like heart rate and its temporal variability, skin temperature, and electrodermal activity can provide general insight into autonomic system activity reflecting stress and adaptive responding to dynamic environments. Such measures have already been encouraged to be included within symptom network analyses (Mansueto et al., 2023; Ralph-Nearman et al., 2024), and appear promising for predicting (hypo)mania states (Gruber et al., 2011). Moreover, these measures can be readily assessed using common tools available commercially. In particular, autonomic data are collected through wearables that are strongly marketed toward the general public. Specifically, ownership ranges between 20 and 40 percent across North America and Europe (European Commission, 2025; Nagappan et al., 2024). Subsequently, with adequate consenting and participant knowledge, these data can be readily indexed by researchers. Furthermore, the additional additional collection of peripheral data includes eye tracking using mobile glasses that track by ocular dynamics and the surrounding environment (Vidal et al., 2012), and decoding visual speech patterns using audio recordings submitted by participants (Berezutskaya et al., 2023). We note that peripheral physiological measures can inform us of neurophysiological activity generally reflecting autonomic arousal in response to affective events, and it alone is not a comprehensive representative of cortical and subcortical dynamics.

### 4.2 Neuroimaging using MRI and neurophysiology

Current idiographic network research has not implemented neurobiological measures, and it is not due to the inherent inability of methods like MRI or EEG to detect minute changes over short timescales. Indeed, repeated scanning over a time period of days, or even minutes, shows that the cortex may undergo rapid reorganization (Wall et al., 2017; Wang et al., 2010). Critically, these findings occurred independently of behavioral or pharmacological interventions, implying a nearly constant evolution of brain structure (i.e., neuroplasticity) that is responsive to typical events through day-to-day life. While these works solely studied neuroanatomy, there is an emerging interest to investigate daily changes to functional neuroimaging measures in conjunction with questionnaire and behavioral batteries. Furthermore, a procedure referred to as *precision functional mapping* that acquires a significant amount of data for each individual, such as hours-long functional MRI, is gaining popularity within clinical contexts through identifying subtle variations to canonical functional network dynamics and topography (Gordon et al., 2017; Lynch et al., 2024), even guiding neuromodulation interventions for a case of intractable major depressive disorder (Nahas et al., 2025). Thus, the existing neuroimaging space appears capable to integrate idiographic methodologies. The remaining discussion will explore particular research avenues, their associated strengths, and limitations, alongside strategies for escaping the impact of limitations on data quality.

Case studies using MRI presented by Wang et al. (2010) and Wall et al. (2017) present the possibility of constructing purely idiographic models for participants that integrate neuroimaging measures. Indeed, a dataset called “Day2Day” is available that consists of approximately 40 sessions of MRI data per individual (*N* = 8) alongside self-report measures (Filevich et al., 2017), which has been used to observe associations between functional connectivity and mood (Racicot et al., 2024). Furthermore, repeated longitudinal imaging reveals the utility of associating mood with intraindividual changes to reward responsiveness indexed by nucleus accumbens BOLD signal throughout multiple sessions (Mattoni et al., 2026). Furthermore, we recognize that the amount of data collection required for these investigations would be taxing for research neuroimaging centers, especially for teams with sole access to a shared neuroimaging center. Resource strain may be mitigated through the creation of multi-site collaborations that harmonize their MRI protocols to increase the number of enrolled participants. However, the financial cost of neuroimaging presents as a significant limiting factor for performing research described in the current perspective article. Some mitigating steps can be made. First, research teams can engage in rapport with their institution’s neuroimaging center to establish a neuroimaging protocol to facilitate after-hours scanning at reduced cost. Indeed, this step led to the production of previous reports of richly sampled neuroimaging datasets by scanning during the night (Gordon et al., 2017; Mattoni et al., 2026). This will allow research teams to collect data that may eventually support grants applications to fund more “traditional” scanning procedures attracting a wider range of potential participants and supporting larger sample sizes. Furthermore, research teams may consider targeting their recruitment toward individuals who are already in frequent contact with medical providers, such as those receiving transcranial magnetic stimulation therapy.

### 4.3 Simultaneous peripheral/neural acquisition

Perhaps one of the most significant opportunities to investigate bioinformed idiographic symptom networks can offered through ambulatory neuroimaging modalities, including wearable scalp EEG, therapeutic stimulation/recording intracranial EEG, and fNIRS. At present, iEEG is an intervention for individuals living with intractable epilepsy to monitor seizure activity and actively interfere with electrical discharges that may initiate a seizure through transient stimulation. Research demonstrates ambulatory iEEG can adequately explore dynamic interactions between neural activity and behavior within ecologically valid environments (Aghajan et al., 2017; Topalovic et al., 2020; Zubair et al., 2026), and can stand to be readily extended to investigate affective paradigms and relationships between neural activity and mood. Recent work demonstrates some psychiatric applications are emerging (Shivacharan et al., 2022). Yet, major limitations include restricted neurobiological scope to subcortical regions and the medial temporal lobe due to their clinical relevance in epilepsy, recruitment difficulty as this intervention is exceedingly rare, and impaired generalizability. Significant spatial coverage is more likely with stereotactic EEG (sEEG) involving surgical implantation of multiple electrodes to identify seizure onset zones to guide further interventions. Neurophysiology data are collected for multiple days, or upward to a month. Ambulatory aspects are limited as individuals must remain proximal to the sEEG amplifier. However, EMA can be implemented to monitor affective and cognitive states. If using methodology similar to Ralph-Nearman et al. (2024), measures of functional connectivity may be estimated to assess global organization as they relate to affective and cognitive responses, or *vice versa*. While this would provide remarkable insights to the underlying neurobiological contributions of dynamic emotion trajectories, this work would poorly generalize to the broader BD population since iEEG and sEEG are predominately used for the primary diagnosis of epilepsy. However, we note that comorbidity between epilepsy and BD is present, thus targeted recruitment is one potential solution to improve the generalizability of findings (Ettinger et al., 2005; Ottman et al., 2011). In addition, emerging research highlights the utility of intracranial interventions within mood disorders like BD, thus targeted investigations recruiting individuals with a primary diagnosis of BD appear likely in the future. However, additional work is necessary as the current intracranial literature for BD appears focused upon depressive episodes (Mutz, 2023).

### 4.4 Idiographic modeling implementation

Current guidelines investigating behavioral symptom networks recommend the investigation of 6 to 8 variables (Levinson et al., 2021; Mansueto et al., 2023). Identifying brain and behavior relationships can easily generate thousands of neural measures per individual. However, there are methods to generate a single measure which represents either structural or functional organization of the brain. For instance, graph theory can represent global connectedness within a single value known as *global efficiency* or the average local-connectedness through *local efficiency* (Sporns, 2013). These values can be applied to many neuroimaging modalities including structural, diffusion, and functional MRI, in addition to fNIRS, EEG and MEG. While this reduces the number of neural measures entered into an idiographic network, this does not necessarily improve the identification of which neurobiological system to investigate (i.e., structure or function). Racicot et al. (2024) targeted high dimensionality by performing principal component analyses on functional connectivity matrices, which often include more than 100 unique connectivity pairs, to minimize the number of potential neurofunctional variables to 30. While this addresses concerns of dimensionality, model overfitting remains a concern when testing models with many predictors, but relatively small sample sizes. Other procedures can be implemented such as Lasso, Ridge, or elastic-net regularization which penalizes of variables in accordance to factors such as multicollinearity and sample size to minimize overfitting. This results in a sparser model possessing greater interpretability and theoretical relevance. This results in a sparser model possessing greater interpretability and theoretical relevance (Racicot et al., 2024). Furthermore, multilevel idiographic network models that include a select number of MRI-derived measures could be completed to determine potential brain-behavior relationships to minimize the effect of exhausting power or overfitting. While multiple models may be significant, metrics such as Akaike or Bayesian information criterion (AIC and BIC, respectively) may be used to select more superior models.

Resulting datasets can provide the opportunity to identify individualized neurobiological predictors of affective, behavioral, and/or cognitive shifts over time, similar to work presented in Ralph-Nearman et al. (2024). However, it is critical to note that idiographic analyses often use multiple samples per day to construct temporal networks. This methodological design from MRI measures is unrealistic (as stated above), instead necessitating sparse experience sampling. While this may impair the ability to detect potential neurobiological predictors of dynamic emotion trajectories, nonetheless it feasible through procedures such as dynamic time warping. As mentioned previously, dynamic interactions between symptom clusters have been observed with just approximately 6 samples within 3–6 months (Mesbah et al., 2024). Furthermore, the robustness of sparse idiographic models can be strengthened by generating individual networks guided by overlapping (i.e., nomothetic) features across the sample. This can be modeled using GIMME (Wright et al., 2019). One particular advantage of GIMME is the ability to model lagged connections, thus researchers can explore the effects of neural measures at timepoint *x* on affective state *n*-days later, and vice versa. Thus, semi-regular neuroimaging sessions seem sufficient for investigating neurobiological interactions with symptom networks. Furthermore, Hidden Markov Modeling, which is already used to model functional MRI, can be used to construct higher-order states possessing a constellation of features which predict future affective states.

## 5 Conclusion

Neurobiological research in BD remains a promising avenue for identifying markers of affective states. Yet, the current literature speaks more toward neurobiological trait features of the condition instead of underlying mechanisms driving affective state transitions between (hypo)mania and depression. Idiographic symptom network analyses may serve as a promising tool for identifying neurobiological predictors of state transitions and improved sensitivity for exploring individualized experiences of mixed states. This tool uses graph theory to construct a network of dynamic symptom interactions over time. As such, imbedding neurobiological measures into symptom networks could provide insights to complex, and likely reciprocal, interactions between cognitive, affective, and neurobiological variables.

## Data Availability

No data or code is associated with this article.
